# Health disparities in Europe’s ageing population: the role of social network

**DOI:** 10.1080/16549716.2018.1445498

**Published:** 2018-03-19

**Authors:** Jenny Olofsson, Mojgan Padyab, Gunnar Malmberg

**Affiliations:** a Centre for Demography and Ageing Research (CEDAR), Umeå University, Umeå, Sweden; b Department of Social Work, Umeå University, Umeå, Sweden; c Department of Geography and Economic History, Umeå University, Umeå, Sweden

**Keywords:** self-rated health (SRH), ageing, Europe, social network, Survey of Health Ageing and Retirement in Europe (SHARE)

## Abstract

**Background**: Previous research suggests that the social network may play very different roles in relation to health in countries with differing welfare regimes.

**Objective**: The study aimed to assess the interplay between social network, socioeconomic position, and self-rated health (SRH) in European countries.

**Methods**: The study used cross-sectional data on individuals aged 50+ from the fourth wave of the Survey of Health, Ageing and Retirement in Europe (SHARE) and includes data from 16 countries. The outcome is poor SRH. All analyses are adjusted for age and stratified by gender.

**Results**: Low satisfaction with the social network was associated with poor SRH among women in all country groups, but predicted poor SRH among males in West/Central and Eastern Europe only. The results from the multivariable analysis showed an increased likelihood of poor SRH among those with relatively lower education, as well as among those with low satisfaction with the social network (women from all country groups and men from Western/Central and Eastern Europe). However, the results from interaction analysis show that poor SRH for those with lower relative position in educational level was greater among those with higher satisfaction with the social network among male and female participants from Northern Europe. The health of individuals who are highly satisfied with their social network is more associated with socioeconomic status in Northern Europe.

**Conclusions**: This study highlights the significance of social network and socioeconomic gradients in health among the elderly in Europe.

## Background

In these times of population ageing and trends of decreasing family size, the density and functionality of social networks is becoming a key issue for health outcome and well-being. This applies to the ageing cohorts throughout Europe, across gender, socioeconomic groups, and countries with different welfare models. It is suggested that social networks may influence health through various mechanisms, including for instance social support, social influence and access to resources [1]. Several studies have revealed a significant influence of the density and functionality of social networks and social isolation on health [2,3], and confirm that well-being and social support are related to the quality and social density of relationships [4–6], perhaps particularly for the older segment of the population [6].

Assuming that individuals’ degree of happiness represents a certain state of emotional well-being, and that interpersonal environment influences emotional well-being, the degree of subjective well-being increases with the number of people constituting the immediate social context [7]. This suggests that the social network, concerning both its quantity and quality, will affect an individual’s degree of subjective well-being and self-rated health (SRH). However, it is obvious that the outcome depends on the character of social contacts and that high frequency of contacts may also have an adverse impact on health [1]. Therefore, we stress the importance of examining the subjective social network satisfaction.

While research on social network and health tend to focus on the meso- to micro-level, Berkman et al., already in a pivotal paper from 2000, stressed the importance of looking also at the macro-level to see how structural conditions shape the social network [1]. They suggested that the structure and function of social networks are shaped by macro-level conditions including culture, socio-economic factors and politics. Thanks to data from the Survey of Health, Ageing and Retirement in Europe (SHARE), we have in this article been able to compare how subjective satisfaction with social networks is associated with SRH in European countries with different socio-economic structures and welfare models, thus linking the macro-, meso- and micro-level in the same study. A key issue is thus how social networks may have a different function in countries characterised by a family based welfare model as in Southern and Eastern Europe as compared to other countries for instance the Scandinavian with a more universal welfare regime and countries in Central and Western Europe with lower levels of public transfers. A crucial question is to what extent social networks has become less decisive for health outcome in countries where the state has taken over most of the support responsibilities.

Prior studies show that there are social class differences in patterns of social interaction and that such patterns vary within cultures by socioeconomic position and gender, and thus may have different effects on health [8]. Cross-national comparisons reveal closer intergenerational contacts in Southern and Eastern Europe, with a family-based welfare model, compared to the Scandinavian universal welfare regime [9]. Even though intergenerational contacts are less frequent in the Scandinavian countries and more old people live alone, several studies report greater satisfaction with the social network as compared to countries in Southern and Eastern Europe [10,11]. Previous studies have also shown that the impact of intergenerational contacts varies across socioeconomic groups and that, for instance, family ties are denser among the less educated and partly compensate for a lack of other resources, even in countries with strong public welfare institutions [12]. Moreover, a great body of research has revealed large gender differences in relation to both subjective health and the importance of social networks [9,13,14]. Hence, we explore the extent to which social network satisfaction – across genders, socioeconomic position (level in the educational hierarchy), and countries with different welfare models – is associated with the subjective well-being of people aged 50+ in the 16 countries participating in SHARE. By conducting cross-country analyses, we consider that the associations between social network satisfaction and SRH may differ depending on different sociocultural contexts and welfare regimes [15,16].

Subjective well-being is an important clinical and societal outcome [17,18]. Measuring the subjective well-being and identifying its determinants among elderly has become a relevant research topic in Europe, owing to an increased life expectancy and growth in the elderly population. The increasing interest in subjective well-being and SRH is also related to the gap between objective determinants of well-being and the layman’s own evaluation of it [17]. However, previous studies indicate that SRH may be a good predictor of health outcomes, considering culture-specific differences [19–21]. Moreover, it has been known that a consistent determinant of poor well-being is socioeconomic position, with those who live through socioeconomic disadvantage experiencing poorer well-being [15,22]. Different dimensions of socioeconomic position – such as education, wealth, and occupational or social class – have been considered in research on social inequalities in health. However, using occupational or social class as measurements of social inequality among the elderly is controversial since not many elderly women have worked. Moreover, the occupational gradient in health decreases over time for older people after leaving the labour market, suggesting occupation as an inappropriate indicator of social class among elderly [23]. Education is a more stable indicator of social class throughout the life-course and can be used as an alternative indicator of social class [24].

Research on the social determinants of health among older people has only recently started integrating different approaches that previously tended to be studied separately: socioeconomic position, family characteristics, and social support [25]. The study’s overall aim is to assess the interplay between social network satisfaction, socioeconomic position, and SRH among elderly men and women in European countries with different welfare regimes. Specific research questions are: (1) the extent to which social network satisfaction is associated with SRH; (2) whether or not relative educational position is associated with SRH; and (3) whether social network satisfaction may moderate the influence of socioeconomic position on poor SRH.

## Methods

### Data

The current cross-sectional study is based on individual level data from release 1.1.1 of the fourth wave of SHARE, collected in 2010–2011 [26]. SHARE is a bi-annual longitudinal survey, focusing on economic and social issues in the ageing population in Europe. The fourth wave consists of 40,129 households (58,489 individuals) surveyed in 16 countries (Austria, Germany, Sweden, the Netherlands, Spain, Italy, France, Denmark, Switzerland, Belgium, the Czech Republic, Poland, Hungary, Portugal, Slovenia, and Estonia). These countries were collapsed into four sub-categories, each representing a welfare state regime (see e.g. [27,28]. Northern Europe (Sweden, Denmark) is characterized by state aid dependence with universal social rights. Western and Central Europe (Germany, Austria, France, Belgium, the Netherlands, Switzerland) belongs to a model with rather low public transfers. Eastern Europe (the Czech Republic, Poland, Hungary, Slovenia, Estonia) formerly belonged to the socialist block and the care system relies to a great extent on family members. Lastly, Southern Europe (Spain, Italy, Portugal) follows a more family based care system.

The sample encompasses 54,751 individuals aged 50 and older.

#### Measures

The outcome variable is SRH, which is a common measure of health in empirical research [29,30], especially when the focus is on assessing the effect of social capital and social network on health [31,32]. A binary variable is created from the original five items (from bad to very good health), whereby 0 is given to individuals reporting good or very good health, and 1 if worse health status is reported.

Education is used as the socioeconomic indicator and is measured on the International Standard Classification of Education Scale (ISCED-97), ensuring that it is internationally comparable between different European countries. It ranges from 0 (low) to 6 (high). ISCED-97 code 6 has not been used in all countries; e.g. in Sweden only five categories are available, with code 5 representing the highest education level.

In order to measure social networks, the fourth wave of the SHARE survey introduced a new social network module employing a direct approach for social network derivation [33]. This social network module uses a name generator for network identification, and compiles a list of important people in the respondent’s life. This list is subjective, and is based on interpersonal ties that are considered to be of the most importance to the respondents and hence reflective of their personal ties. The interviewer asks a direct probing question ‘Over the last 12 months, who are the people with whom you most often discussed important things?’ The respondent is asked to name up to seven persons as their network members. These network members are meaningful people that they trust and share things of relative importance. The respondents’ social network was measured in terms of their satisfaction with it. The satisfaction variable is derived from two questions distinguishing between respondents with and without cited social network members. Respondents with cited social networks members answered the question: ‘Overall, how satisfied are you with the relationship that you have with the person(s) we have just talked about? Please answer on a scale from 0 to 10 where 0 means completely dissatisfied and 10 means completely satisfied?’ and respondents without confidants answered the question: ‘You indicated that there is no one with whom you discuss matters, and no one who is important to you for some other reason. How satisfied are you with this on a scale of 0–10, where 0 means completely dissatisfied and 10 means completely satisfied?’ The derived network satisfaction variable collapses two variables into one overall measure of the respondent’s social network satisfaction ranging from 0 to 10. Satisfaction with social network was dichotomized by the median scores within each gender and country group. The dichotomized variables was then used in the exploration of interactions.

### Statistical analysis

The proportion of poor SRH within the categories of sociodemographic characteristics and social network was compared by means of chi-square test.

The socioeconomic status of an educational group is conceptualized as the group’s relative position in the social hierarchy. The relative educational position indicator is a value between 0 (most disadvantaged) and 1 (most advantaged), quantified using the relative position in the cumulative population distribution of the midpoint in each category within the educational hierarchy [34] and used to calculate the relative index of inequality (RII). This measure makes use of the information on the health outcome in every socioeconomic status category, rather than simply comparing the extreme groups at the top and bottom. It also seemed crucial to focus not only on the absolute socioeconomic status but also on the relative position, since education has different proportions in the population in each country group and the ratios could not be directly compared. The RII is used in order to make valid comparisons [34]. The relative educational position indicator is calculated as the proportion of the population that has a higher position in the social hierarchy. For example, the highest educational group comprises 1.1% of the male population in Southern Europe. The relative position of its members would be between 0 and 0.011, the average being 0.0055. The next most favourable category represented 12.6% of the population and for the mid-point person a proportion of 6.3% of the population in this category would be above them. All 1.1% with the higher educational level were taken to be in more favourable circumstances than this group, and the RII score for this group would thus be 1.1 plus 0.063. This procedure is then continued for the rest of the educational categories, separately for each country group and gender. The educational position indicator was considered as an independent variable in our analyses, and was related to poor SRH in multivariable logistic regressions. The regression coefficients of the relative educational position indicator and the standard error were used to calculate the probability of poor SRH, and can be interpreted as the probability of poor SRH moving from the top (0) to the bottom (1) of the educational hierarchy. The larger the probability, the greater the degree of inequality of poor health across the socioeconomic (education) hierarchy. In other words, higher probability indicates an increased likelihood of reporting poor SRH when the lowest educational category is compared with the highest. Two models were used, one adjusted for age (Model 1) and one adjusted for age and social network satisfaction (Model 2). Further, we examined the interaction effect between educational position indicator and social network satisfaction using the factorial interaction in the regression model. If the interaction was significant, a marginal effect of relative educational position and an incremental effect of social network satisfaction on poor SRH were estimated in the predicted probability metric, taking into account the interaction term. All analyses are conducted in each country group and stratified by gender. Statistical analyses were done with Stata version 13.1 (StataCorp, College Station, TX). Statistical significance was defined as p < 0.05.

## Results

There were 54,751 individuals aged 50 and older included in the analysis of whom 56% (n = 30,808) were women and 44% (n = 23,943) were men (Table 1). The mean age for the total population was 66,2 ± 9,9 years (66 ± 10 in Western & Central, 67 ± 10 in Northern, 66 ± 10 in Southern, and 66 ± 10 in Eastern). The population was distributed equally across gender regarding age.

Table 2 shows summary statistics over the distribution characteristics of the sample. The results show that the proportion of those reporting poor SRH was lower in the younger age groups, and this applies to both men and women across all country groups. In total, there was a significantly higher proportion of poor SRH among those with low satisfaction with their social network compared to individuals with high social network satisfaction (Table 2). For men, a higher proportion of individuals reported poor SRH among those with a smaller size of network than did those with an extended network (42.0% vs. 38.0%, p < 0.001). This association was confirmed only among male participants from West and Central Europe. However, the proportion of poor SRH was not significantly different among women with a small social network compared to those with an extended social network (44.2% vs. 43.7%, Table 2). That is why in further analysis and multivariable models, only satisfaction with social network is included.

**Table 1.** Distribution (%) of characteristics of the participants by country and sex.

Men (n = 23,943)	North EU	West and Central EU	East EU	South EU	Total
Age in years					
50–59	24.7	30.8	30.4	25.7	29.4
60–69	36.7	35.3	36.4	34.3	35.6
70–79	25.7	23.4	23.9	28.2	24.5
80–89	11.5	9.7	8.9	10.7	9.7
90 and more	1.5	0.8	0.4	1.1	0.8
*Education (ISCED)*					
0 (Low)	0.05	2.3	0.58	6.9	2.3
1	18.6	12.8	7.5	42.4	16.2
2	9.7	12.5	24.8	20.8	17.8
3	35.1	39.8	42.7	15.2	36.5
4	3.7	4.4	6.4	1.0	4.5
5	32.9	26.5	17.4	12.6	21.7
6 (High)	0	1.7	0.5	1.1	1.1
*Self-rated health*					
Good or very good	76	71	46	57	60
Poor	24	29	54	43	40
*Social network*					
Size in scale 0–7 (Mean±sd)	2.3 ± 1.5	2.5 ± 1.6	2.0 ± 1.3	2.3 ± 1.5	2.3 ± 1.5
Satisfaction in scale 0–10 (Mean±sd)	9.1 ± 1.3	8.6 ± 1.4	8.7 ± 1.6	8.8 ± 1.5	8.8 ± 1.5
Women (n = 30,808)					
Age in years					
50–59	25.7	32.1	29.7	29.6	30.4
60–69	36.2	32.8	34.1	33.5	33.6
70–79	23.3	22.1	24.6	24.4	23.4
80–89	12.4	11.5	10.7	11.1	11.2
90 and more	2.3	1.6	0.9	1.5	1.4
*Education (ISCED)*					
0 (Low)	0.09	3.2	0.9	9.9	3.2
1	20	17.7	13.6	47.9	21.0
2	13.1	19.3	24.6	18.0	20.6
3	26.2	35.8	37.5	11.5	32.1
4	4.4	2.8	8.4	1.4	4.7
5	36.3	20.2	14.7	10.9	17.9
6 (High)	0	1.0	0.3	0.4	0.6
*Self-rated health*					
Good or very good	72	68	42	49	56
Poor	28	32	58	51	44
*Social network*					
Size in scale 0–7 (Mean±sd)	2.9 ± 1.6	2.9 ± 1.7	2.4 ± 1.5	2.5 ± 1.5	2.6 ± 1.6
Satisfaction in scale 0–10 (Mean±sd)	9.2 ± 1.3	8.8 ± 1.3	8.8 ± 1.6	8.9 ± 1.4	8.8 ± 1.4

**Table 2.** Percentage (%) of poor SRH within categories of sociodemographic characteristics and social network, by country and gender.

Men	West and Central EU	North EU	South EU	East EU	Total
Age in years					
50–59	23***	16***	26***	43***	30***
60–69	25	19	41	51	36
70–79	34	29	50	66	47
80–89	48	42	64	75	59
90 and more	59	48	71	76	64
*Education (ISCED)*					
0 (Low)	54***	-	62***	66***	59***
1	46	39***	52	66	52
2	34	31	35	64	48
3	29	22	26	50	36
4	14	19	28	61	38
5	19	16	35	43	27
6 (High)	25	-	25	45	28
*Social network: size*					
Small	32.5***	24.0	43.0	53.1	42.0***
Extended	27.5	24.0	42	55.7	38.0
*Social network: Satisfaction*					
Low	30.9***	25.1	44.9	57.6***	40.8**
High	27.8	23.1	41.6	52.7	38.7
Women					
Age in years					
50–59	23***	19***	35***	44***	32***
60–69	26	23	46	52	38
70–79	39	33	64	72	55
80–89	51	47	76	80	64
90 and more	52	52	69	82	62
*Education (ISCED)*					
0 (Low)	61***	-	77***	67***	69***
1	47	41***	59	69	56
2	34	38	40	69	50
3	27	28	29	52	38
4	15	32	36	61	46
5	22	17	38	41	28
6 (High)	19	-	26	44	25
*Social network: size ^b^*					
Small	35.5***	31.2*	55.4***	56.9	44.2
Extended	28.4	25.8	49.8	58.3	43.7
*Social network: satisfaction ^b^*					
Low	32.9**	31.0*	54*	60.4***	44.8*
High	30.7	26.3	50	56.6	43.5

*Note*: b Size and satisfaction of the social network are dichotomized by median within each country subgroup

*p < 0.05 ** p < 0.01 *** p < 0.001

The proportion of poor SRH was highest among those in the lowest educational category compared to the highly educated individuals across all country groups (Table 2). This finding was more pronounced among women, with 69% in the lowest educational category reporting poor SRH compared to 59% among men (Table 2). The results of the multivariable logistic regressions including relative educational position showed that, when adjusted for age, the predicted probability of poor SRH is 0.26 greater among those in the lowest educational category compared to the highest (predicted probability = 0.2598, p < 0.001, Table 3). In other words, one unit shift from higher to lower education results in a 26-percentage-point increase in the probability of reporting poor SRH. After controlling for satisfaction with the social network, the probability of poor SRH decreased by 0.04 percentage point (0.2598 to 0.2594) among men (Table 3).

**Table 3.** Predicted probability of poor SRH, by country group.

Men	West and central EU	North EU	South	East	Total
*Model 1*					
Relative Educational position	0.2835***	0.2245***	0.2629***	0.2349***	0.2598***
*Model 2*					
Relative Educational position	0.2818***	0.2252***	0.2648***	0.2332***	0.2594***
Social network satisfaction	−0.026**	−0.031	−0.0323	−0.0500***	−0.020***
Women					
*Model 1*					
*Relative Educational position*	0.2614***	0.2323***	0.3364***	0.2125***	0.2497***
*Model 2*					
Relative Educational position	0.2612***	0.2365***	0.3411***	0.2135***	0.2504***
Social network satisfaction	−0.025**	−0.052**	−0.038**	−0.035***	−0.0146**

*p < 0.05; ** p < 0.01; *** p < 0.001

Model 1. Adjusted for age.

Model 2. Adjusted for age+ social network satisfaction.

Among female respondents, a protective effect of high social network satisfaction was found (Figure 2), and those who were highly satisfied were 1.5 percentage points less likely to report poor SRH (Table 3). The protective effect of high social network satisfaction was highest among female respondents from Northern Europe (5.2 percentage points less likely to report poor SRH, Table 3).

A stratified analysis by country group shows a null effect of social network satisfaction on poor SRH among men in Northern and Southern Europe (Table 3). However, a protective effect of high satisfaction with the social network was found among male respondents from Eastern Europe, whereby highly satisfied individuals were approximately 5 percentage points less likely to report poor SRH (Table 3).

**Figure 1.**
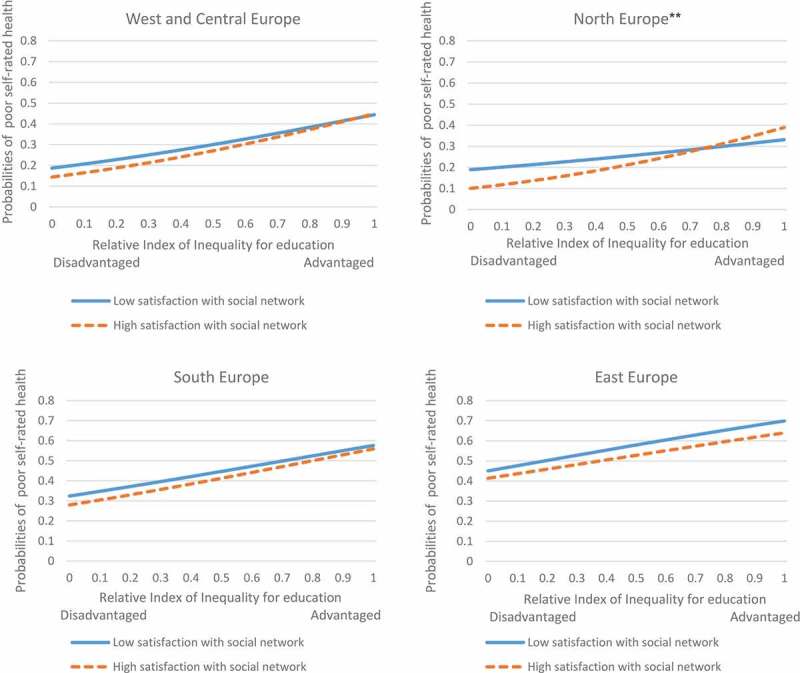
Probabilities of poor SRH by satisfaction with the social network among males in different country groups. **p < 0.01 for the interaction effect.

A similar pattern with slightly lower likelihood was found among male respondents from Western and Central Europe, revealing a 2.6-percentage-point lower probability of reporting poor SRH among those with lower satisfaction with their social network compared to those with higher satisfaction (Table 3). A significant interaction between relative educational position and social network satisfaction was found among male respondents from Northern Europe (Figure 1), where the probability of reporting poor SRH corresponding to one unit shift from higher to lower educational level was approximately double among those with high social network satisfaction than those with low satisfaction (28 vs. 14 percentage points, respectively).

**Figure 2.**
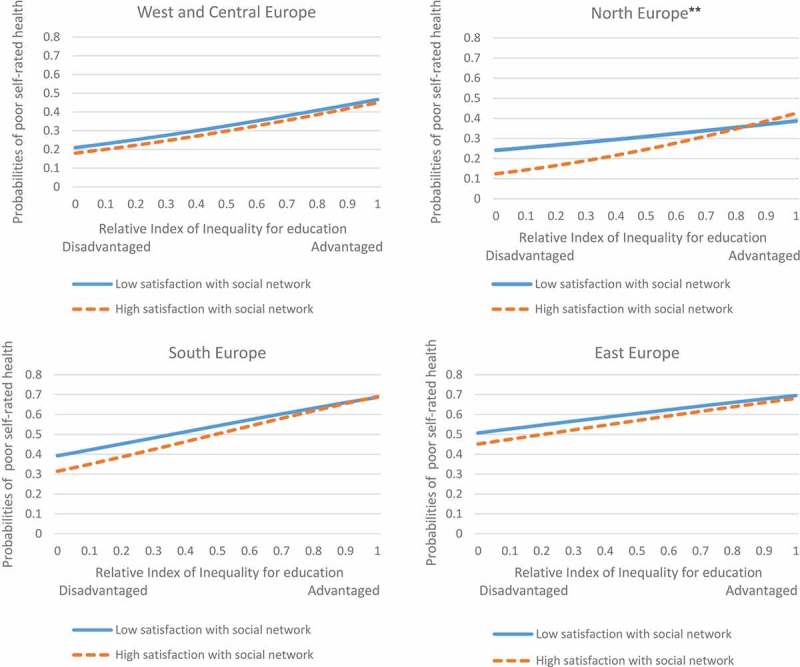
Probabilities of poor SRH by satisfaction with the social network among females in different country groups. **p < 0.01 for the interaction effect.

A significant interaction effect between relative educational position and satisfaction with the social network among female respondents in Northern Europe (Figure 2) suggests a higher probability of poor SRH for lower-educated individuals among those who are highly satisfied with their network compared to less satisfied respondents (29 vs. 14 percentage points, respectively).

## Discussion

The aim of this study was to describe the social network among men and women in old age from 16 European countries and its moderating role regarding the socioeconomic differential in poor SRH. By analysing impact of social network satisfaction (meso-level) on SRH (micro-level) in 16 European countries with different welfare regimes (macro-level) we follow the framework suggested by Berkman et al [1], suggesting that the influence from social networks on health should be analysed in relation to socio-economic and political conditions at macro-level.

In summary, the study suggests three key findings responding to the research questions. First, this study provides evidence on the association between satisfaction with the social network and SRH among the elderly. The protective effect of social network was considerable among females in general, and more specifically among females in Northern Europe. This indicates that social networks are more important for the well-being of women, and is in line with findings showing that women are more active in maintaining social contacts, especially within the family [35]. The results indicate that satisfaction with the social network is more correlated with good SRH in countries in the North. One possible explanation for this could be that social networks become more important for people’s well-being in countries where family-based support is less common and is not taken for granted. Moreover, a higher satisfaction with social networks in Northern European countries could be because they do not have to rely much on their network confidents owing to the greater role of the state in their welfare as compared to countries with more family based welfare models.

Previous studies based on SHARE have also shown that elderly old people in the Scandinavian countries are more satisfied with their social network despite less frequent contact [9,10], and report less loneliness despite a higher prevalence of living alone and other indicators of assumed social community [11]. One explanation could be that in the universal welfare regime the family may have a complementary and more positive role in addition to the more daily-based support from public providers.

Second, socioeconomic inequalities in the poor SRH of individuals in old age were found on country level across all welfare regimes, but with different magnitudes. The greatest educational inequality in SRH was found among women in Southern Europe whereas the narrowest inequality was found among women in Eastern Europe, followed by men in Northern Europe. Similar studies with a specific focus on quality of life have been conducted using SHARE data [16,36], in which the observed differences in quality of life between those with and without a limited illness, as well as between the least and most educated individuals, are reported. Using RII is appropriate, as it measures the total impact of education inequalities on poor SRH of the total population. The main difference between the effect of low education on poor SRH and the total impact of educational inequality on poor SRH is that the measure of total impact accounts for both the effect of decreased education on the health outcome, as well as educational inequalities in a certain population by taking into account the number of individuals in each educational category. The greater the extent of inequalities in education, the higher the measures of total impact will be. In other words, this approach assumes that the effect of one year of education on mental health is greater if the differences in education level between the upper and lower levels are larger. Similar to our findings, poor quality of life is explained to a greater extent by lower education than having limited illness in Greece, Italy and Spain [16].

Third, while our research on the interaction between socioeconomic position and type of welfare regime revealed few significant results, we found an association between poor SRH and high satisfaction with the social network among lower-educated individuals in Northern Europe, which was more pronounced among men. While prior research has provided empirical evidence on the effect of social network on health among older Europeans [37], our unique contribution lies in the role of education and gender on such an association. We speculate that this could be explained by reverse causality [13,37,38], suggesting that people with lower health status are more likely than their healthier counterparts to need and seek help from their social networks. This effect is more highlighted among the lower-educated elderly in Northern Europe in the current analysis because they demand more resources than do higher-educated individuals, and also because they have the resources to do so thanks to the welfare regimes there. Another argument could be the fact that not all social contacts support and foster well-being. Social networks can sometimes be perceived as emotionally demanding and thus become stressful, unwanted and unpleasant and may, potentially, result in worse health [13].

Noteworthy to mention that the data used in this analysis are cross-sectional and therefore we cannot identify the causal mechanism underlying the association between poor SRH and higher social network satisfaction among lower-educated individuals in Northern Europe. Another limitation is utilizing self-reported questionnaires. Even though, self-reported instruments are rather subjected to reflects one’s general perception of health, they are useful tools to screen and monitor health condition at the population level. The issue of self-reported questionnaires may arise another source of limitation regarding cross-national comparability and that interpretation and understanding of questions can vary in different socio-cultural contexts. However, SHARE is strictly harmonized and is implemented in the same way across all countries.

Chemaitelly et al. [39] highlight the importance of gender in analysing the significance of social support and networks in women’s and men’s SRH. That gender plays an important role is also shown in this study, as the gap in poor SRH between high and low social network satisfaction was larger among men than women in Northern Europe. A gender-stratified study on the association between social network, group belonging, and collective self-esteem shows that males had more likelihood for reporting negative collective self-esteem that females, whereas females were more likely to report high positive collective self-esteem and were therefore more satisfied with their social network and thus had better general health [40].

Other key findings in this study are the cross-country differentials observed. This shows show the importance of linking the meso- and micro-level to conditions at the macro-level. Berkman et al. have suggested that the causal connection and the social network structure is influenced by conditions at the macro-level, including culture, socioeconomic conditions and politics and mediated by factors such as social support and material resources [1]. In this study we have compared countries with different welfare regimes and found significant differences. However, the country-specific results may due to other socio-economic, cultural and socio-economic country differentials. Hence a key topic for future research would to analyse how the patterns observed are associated with various country-specific policies and socio-economic conditions.

## Conclusion

In conclusion, socioeconomic differentials in health and the effect of network satisfaction on poor SRH vary across European countries with different welfare regimes and differ by gender, which parallels other findings on health and social support [39]. Our results allow us to add valuable knowledge regarding the importance of the association between social relations and SRH in different cultural contexts.

## Paper context

The socioeconomic gradient in health inequality was studied considering a unique contribution of social network, welfare state and gender. Social network satisfaction has a protective effect on health, particularly among women. We found an interaction between socioeconomic status and satisfaction with the social network among elderly from Northern Europe. We recommend gender-stratified policies targeting a reduction in socioeconomic-related health inequality, for which the importance of social network and differences in welfare state regimes is considered.

## References

[1] LF Berkman, Glass T, Brissette I, et al From social integration to health: Durkheim in the new millennium. Soc Sci Med. 2000;51:843–10. 10972429 10.1016/s0277-9536(00)00065-4

[2] Pantell M, Rehkopf D, Jutte D, et al Social isolation: a predictor of mortality comparable to traditional clinical risk factors. Am J Public Health. 2013;103:2056–2062. 24028260 10.2105/AJPH.2013.301261 PMC3871270

[3] Yang YC, McClintock MK, Kozloski M, et al Social isolation and adult mortality: the role of chronic inflammation and sex differences. J Health Soc Behav. 2013;54:182–202. 10.1177/0022146513485244 PMC3998519 23653312

[4] Diener E, Seligman MEP. Very happy people. Psychol Sci. 2002;13:81–84. 11894851 10.1111/1467-9280.00415

[5] Vaux A Social support: theory, research, and intervention. New York: Praeger publishers; 1988.

[6] Shankar A, Rafnsson SB, Steptoe A Longitudinal associations between social connections and subjective wellbeing in the English longitudinal study of ageing. Psychol Health. 2015;30:686–698. 25350585 10.1080/08870446.2014.979823

[7] Requena F Welfare systems, support networks and subjective well-being among retired persons. Social Indic Res. 2010;99:511–529.

[8] Grundy E, Sloggett A Health inequalities in the older population: the role of personal capital, social resources and socio-economic circumstances. Soc Sci Med. 2003;56:935–947. 12593868 10.1016/s0277-9536(02)00093-x

[9] Hank K Proximity and contacts between older parents and their children: a European comparison. J Marriage Family. 2007;69:157–173.

[10] Olofsson J, Malmberg G Äldre europeérs sociala nätverk In: Fors F, Olofsson J, editors. Utblick – Sverige i en internationell jämförelse. Umeå: Sociologiska institutionen, Umeå universitet; 2016 p. 63–78.

[11] Sundstrom G, Fransson E, Malmberg B, et al Loneliness among older Europeans. European. J Ageing. 2009;6:267–275. 28798610 10.1007/s10433-009-0134-8 PMC5547349

[12] Ulmanen P, Szebehely M From the state to the family or to the market? Consequences of reduced residential eldercare in Sweden. Int J Soc Welf. 2015;24:81–92.

[13] Pinquart M, Sorensen S Influences of socioeconomic status, social network, and competence on subjective well-being in later life: A meta-analysis. Psychol Aging. 2000;15:187–224. 10879576 10.1037//0882-7974.15.2.187

[14] McLaughlin D, Vagenas D, Pachana NA, et al Gender differences in social network size and satisfaction in adults in their 70s. J Health Psychol. 2010;15:671–679. 20603290 10.1177/1359105310368177

[15] Litwin H Social networks and self-rated health - A cross-cultural examination among older Israelis. J Aging Health. 2006;18:335–358. 16648390 10.1177/0898264305280982

[16] Niedzwiedz CL, Katikireddi SV, Pell JP, et al Socioeconomic inequalities in the quality of life of older Europeans in different welfare regimes. Eur J Public Health. 2014;24:364–370. 24568754 10.1093/eurpub/cku017 PMC4032483

[17] Stiglitz J, Sen A, Fitoussi J-P Report by the commission on the measurement of economic performance and social progress. Paris: The Commission; 2010.

[18] Diener E, Chan MY Happy people live longer: subjective well‐being contributes to health and longevity. Appl Psychol Health Well‐Being. 2011;3:1–43.

[19] Emmelin M, Weinehall L, Stegmayr B, et al Self-rated ill-health strengthens the effect of biomedical risk factors in predicting stroke, especially for men - an incident case referent study. J Hypertens. 2003;21:887–896. 12714862 10.1097/00004872-200305000-00012

[20] Jürges H True health vs response styles: exploring cross‐country differences in self‐reported health. Health Econ. 2007;16:163–178. 16941555 10.1002/hec.1134

[21] Jylhä M What is self-rated health and why does it predict mortality? Towards a unified conceptual model. Soc Sci Med. 2009;69:307–316. 19520474 10.1016/j.socscimed.2009.05.013

[22] Knesebeck OV, Wahrendorf M, Hyde M, et al Socio-economic position and quality of life among older people in to European countries: results of the SHARE study. Ageing Soc. 2007;27:269–284.

[23] Hyde M, Jones IR The long shadow of work—does time since labour market exit affect the association between socioeconomic position and health in a post-working population. J Epidemiol Commun H. 2007;61:533–539. 10.1136/jech.2006.051284 PMC2465706 17496263

[24] Arber S, Cooper H Gender and inequalities in health across the lifecourse In: Annandale E, Hunt K, editors. Gender inequalities in health. Milton Keynes: Open University Press; 2000 p. 123–149.

[25] Rueda S, Artazcoz L Gender inequality in health among elderly people in a combined framework of socioeconomic position, family characteristics and social support. Ageing Soc. 2009;29:625–647.

[26] Börsch-Supan A Survey of health, ageing and retirement in Europe (SHARE) wave 4. Release Version: 1.1.1 SHARE-ERIC. Data set, 2013. 10.1093/ije/dyt088 PMC3780997 23778574

[27] Esping-Andersen G The three worlds of welfare capitalism. Cambridge: John Wiley & Sons; 2013.

[28] Bambra C Health inequalities and welfare state regimes: theoretical insights on a public health ‘puzzle’. J Epidemiol Commun H. 2011;65:740–745. 10.1136/jech.2011.136333 21690243

[29] Blakely TA, Lochner K, Kawachi I Metropolitan area income inequality and self-rated health - a multi-level study. Soc Sci Med. 2002;54:65–77. 11820682 10.1016/s0277-9536(01)00007-7

[30] Craig N Exploring the generalisability of the association between income inequality and self-assessed health. Soc Sci Med. 2005;60:2477–2488. 15814173 10.1016/j.socscimed.2004.11.018

[31] Kawachi I, Kennedy BP, Glass R Social capital and self-rated health: A contextual analysis. Am J Public Health. 1999;89:1187–1193. 10432904 10.2105/ajph.89.8.1187 PMC1508687

[32] Eriksson M, Ng N, Weinehall L, et al The importance of gender and conceptualization for understanding the association between collective social capital and health: A multilevel analysis from northern Sweden. Soc Sci Med. 2011;73:264–273. 21689877 10.1016/j.socscimed.2011.05.013

[33] Litwin H, Stoeckel K, Roll A, et al Social network measurement in SHARE wave four. In: Malter F, Börsch-Supan A, editors. SHARE wave 4: innovations and methodology. Munich: MEA, Max Planck Institute for Social Law and Social Policy; 2013 p. 18–38.

[34] Naess Ø, Claussen B, Thelle DS, et al Cumulative deprivation and cause specific mortality. A census based study of life course influences over three decades. J Epidemiol Commun H. 2004;58:599–603. 10.1136/jech.2003.010207 PMC1732810 15194723

[35] Cornwell B Independence through social networks: bridging potential among older women and men. J Gerontol B-Psychol. 2011;66:782–794. 10.1093/geronb/gbr111 PMC3198249 21983039

[36] Blane D, Netuveli G, Montgomery SM Quality of life, health and physiological status and change at older ages. Soc Sci Med. 2008;66:1579–1587. 18241966 10.1016/j.socscimed.2007.12.021

[37] Deindl C, Hank K, Brandt M Social networks and self-rated health in later life In: Börsch-Supan A, Brandt M, Litwin H, et al., editors. Active ageing and solidarity between generations in Europe: first results from SHARE after the economic crisis. Boston: De Gruyter; 2013 p. 301–309.

[38] Sirven N, Debrand T Social participation and healthy ageing: an international comparison using SHARE data. Soc Sci Med. 2008;67:2017–2026. 18973973 10.1016/j.socscimed.2008.09.056

[39] Chemaitelly H, Kanaan C, Beydoun H, et al The role of gender in the association of social capital, social support, and economic security with self-rated health among older adults in deprived communities in Beirut. Qual Life Res. 2013;22:1371–1379. 23011493 10.1007/s11136-012-0273-9

[40] Barker V Older adolescents’ motivations for social network site use: the influence of gender, group identity, and collective self-esteem. CyberPsychol Behav. 2009;12:209–213. 19250021 10.1089/cpb.2008.0228

